# The Contribution of Basic Psychological Need Satisfaction to Psychological Well-Being via Autonomous Motivation Among Older Adults: A Cross-Cultural Study in China and France

**DOI:** 10.3389/fpsyg.2021.734461

**Published:** 2021-11-04

**Authors:** Minmin Tang, Dahua Wang, Alain Guerrien

**Affiliations:** ^1^School of Psychology, Sichuan Normal University, Chengdu, China; ^2^Univ. Lille, ULR 4072 - PSITEC - Psychologie: Interactions Temps Émotions Cognition, Lille, France; ^3^Institute of Developmental Psychology, Beijing Normal University, Beijing, China

**Keywords:** basic psychological need satisfaction, motivation, psychological well-being, the elderly, cross-cultural

## Abstract

According to self-determination theory (SDT), the satisfaction of the universal needs for autonomy, competence, and relatedness is important in order to enhance autonomous motivation, which in turn promotes psychological well-being (PWB), regardless of age or culture. In contrast, some cross-cultural perspectives challenge SDT's universalistic viewpoint, especially SDT's view that autonomy yields universal positive effects across Western and Eastern societies. To test these theoretical frameworks across cultures, with special focus on the field of aging, this study examined the contribution of satisfying basic psychological needs to elderly people's PWB from Eastern and Western cultures (China and France). Elderly retired people living at home (*N*_China_ = 510, *M*_age_ = 68.49 years; *N*_France_ = 170, *M*_age_ = 71.19 years) were invited to complete surveys assessing these variables and providing demographic information. Consistent with the hypothesis of SDT, results from structural equation modeling (SEM) indicated that needs satisfaction facilitates autonomous motivation, which in turn promotes PWB in both elderly Chinese and French. Moreover, the finding from subsequent moderation analysis confirmed the moderating effect of culture in the relationship between competence satisfaction and PWB, with the contribution of competence satisfaction on PWB being stronger among elderly Chinese than elderly French (*p* < 0.05). Our findings suggest that, the broad applicability of SDT notwithstanding, attending to cultural differences in elderly care remains important.

## Introduction

The proportion of older peoples in the world's population is growing. According to the results of the 2017 Revision of World Population Prospects published by the United Nations, the number of people aged 60 or older is estimated to be 962 million worldwide, comprising 13% of the global population; by 2050, the global population of older people is projected to be 2 billion, or 21% of the world population (United Nations Department of Economic Social Affairs, 2017). In some countries, such as China and France, this situation is even more prominent. According to the National Bureau of Statistics of China, Chinese people aged 60 or over represented 17.3% of the Chinese population in 2016 and this is projected to be 34.1% in 2050. For France, people 60-years-old and over represented 24.6% of the French population in 2015 and this is projected to be 32% in 2050 (INSEE, 2017). Societal issues around older people are thus becoming increasingly important, and naturally include mental health problems. In this global context it is therefore important to understand the determinants of well-being, particularly within mental health promotion in older age. Self-determination theory (SDT) is one of the most important perspectives that focuses on promoting well-being, namely through the satisfaction of the basic psychological needs for autonomy, competence, and relatedness (Ryan and Deci, 2000, 2017). However, the question remains as to the universality of the determinants of well-being among older adults: are they identical or different across cultures? Based on SDT, the present research sets out to improve our understanding of the factors contributing to elderly people's well-being within a cross-cultural perspective.

### Well-Being

Although well-being is central to positive psychology, its definition remains complex and controversial, as it covers concepts from various theories, often of philosophical origin. From a philosophical point of view, the conceptual models and the theoretical frameworks of well-being can be summarized in two approaches: the hedonic approach, which centers on experiences of personal happiness (e.g., Hobbes, Marquis de Sade, Bentham); and the eudaimonic approach, which focuses on the meaning of life and self-realization (e.g., Aristotle). Based on two different philosophical ideas, current research of positive psychology on well-being follows two orientations: subjective well-being (SWB) and psychological well-being (PWB). Subjective well-being is based on the idea of the hedonic approach, which defines well-being in terms of pleasure vs. pain and the three classic assessment indicators are positive emotions, negative emotions, and life satisfaction (e.g., Diener and Lucas, 1999; Kahneman, 1999). Psychological well-being is based on the idea of the eudaimonic approach, which defines well-being in terms of the degree to which a person is fully functioning, and assessment indicators are self-esteem, meaning of life, personal growth, etc. (e.g., Ryff and Singer, 1998).

### The Self-Determination Theory Perspective

Self-determination theory is another perspective that embraces self-realization as a central, defining aspect of well-being, and attempts to explain the developmental pathway for promoting well-being, one which is particularly concerned with basic psychological needs satisfaction and autonomous motivation (Ryan and Deci, 2000, 2017). In addition, SDT posits that fulfillment of these basic psychological needs typically facilitates SWB as well as PWB (Ryan and Deci, 2001). Thus, in SDT research, researchers have typically used SWB as one of several indicators of well-being, and added other indicators from the eudemonic approach such as psychological adjustment, personal growth, meaning of life, self-esteem, subjective vitality, adaptation, purpose, and growth, etc. (e.g., Philippe and Vallerand, 2008; Mackenzie et al., 2017), or employed an holistic perspective scale such as the Psychological Well-Being Manifestations Measure Scale (PWBMMS) (e.g., Paterson, 2007; Sharma, 2013; Indoumou Peppe et al., 2018, 2020), which includes the SWB orientation (e.g., happiness) and also the PWB orientation (e.g., self-esteem) (Massé et al., 1998).

#### Basic Psychological Needs Satisfaction and Psychological Well-Being

According to SDT, three basic psychological needs are distinguished, which are universally important for psychological growth and PWB. The need for autonomy refers to experiencing a sense of volition, congruency, and integration, and cannot be equated with independence (Ryan and Deci, 2017). The need for competence refers to the individual's feeling of effective control and mastery over his social environment and outcomes (Ryan and Deci, 2001; Deci and Ryan, 2002). Finally, the need for relatedness is defined as an individual's need to feel a secure sense of belonging and connection to others in his or her social environment (Ryan and Deci, 2001; Deci and Ryan, 2002). Self-determination theory strongly supports the view that the fulfillment of basic psychological needs contributes to greater psychological health and PWB, such as life satisfaction, positive affect, and vitality (e.g., Sheldon et al., 2004, 2009; Church et al., 2013; Chen et al., 2015; Cordeiro et al., 2016). In contrast, the frustration of basic psychological needs produces a variety of negative consequences such as symptoms of anxiety, depression, or negative affect (e.g., Cordeiro et al., 2016).

#### Motivational Regulation and Psychological Well-Being

Self-determination theory further proposes the existence of different types of motivation. Depending on the degree of “self-determination,” which refers to the experience of freedom in initiating one's behavior, SDT considers motivation on a continuum from amotivation through extrinsic to intrinsic motivation.

Amotivation, which corresponds to non-regulation, refers to the lack or absence of extrinsic or intrinsic motivation. Controlled extrinsic motivation or non-self-determined extrinsic motivation, meaning an individual's behavior depends directly on the external contingencies or internal affective pressures, is less volitional and does not represent true acceptance or true self-regulation—in other words, the behavior is controlled. Autonomous extrinsic motivation or self-determined extrinsic motivation, which means the source and the reason of behavior is instrumental, but derived from a belief in the personal importance or perceived value of the activity, is more volitional, fully accepting: in other words, the behavior is autonomous (Deci and Ryan, 2008). Intrinsic motivation, which corresponds to intrinsic regulation, means that activities are performed with pleasure, interest, or satisfaction (Vallerand and Grouzet, 2001). With a degree of autonomous self-regulation, both intrinsic motivation and autonomous extrinsic motivation are classified as autonomous motivation/self-determined motivation, an entirely volitional form of motivation whereby the individual experiences volition and is wholly willing to engage in the behavior (Ryan and Deci, 2017).

With reference to SDT, autonomous motivation should facilitate PWB, while non-autonomous forms of motivation usually produce psychological dysfunction and the most negative psychological consequences. More specifically, when an individual's behavior is more regulated through autonomous motivation (intrinsic motivation and autonomous extrinsic motivation), they will display a higher quality of behavior and persist for longer at an activity (Senécal et al., 2000); they will also have more positive experiences and derive greater psychological health and PWB (Ryan and Deci, 2000, 2017; Niemiec et al., 2006; Knafo and Assor, 2007). In contrast, when an individual's behavior is more regulated through non-autonomous motivation (amotivation or controlled extrinsic motivation), a variety of negative consequences may result such as more depressive symptoms and less psychological adaptation, self-esteem, life satisfaction, etc. (Benedetti et al., 2015; Rahman et al., 2015; Zuroff et al., 2017; Gillet et al., 2018; Vaquero Solís et al., 2019).

#### Basic Psychological Needs Satisfaction, Motivational Regulation, and Psychological Well-Being

**Figure 1:**
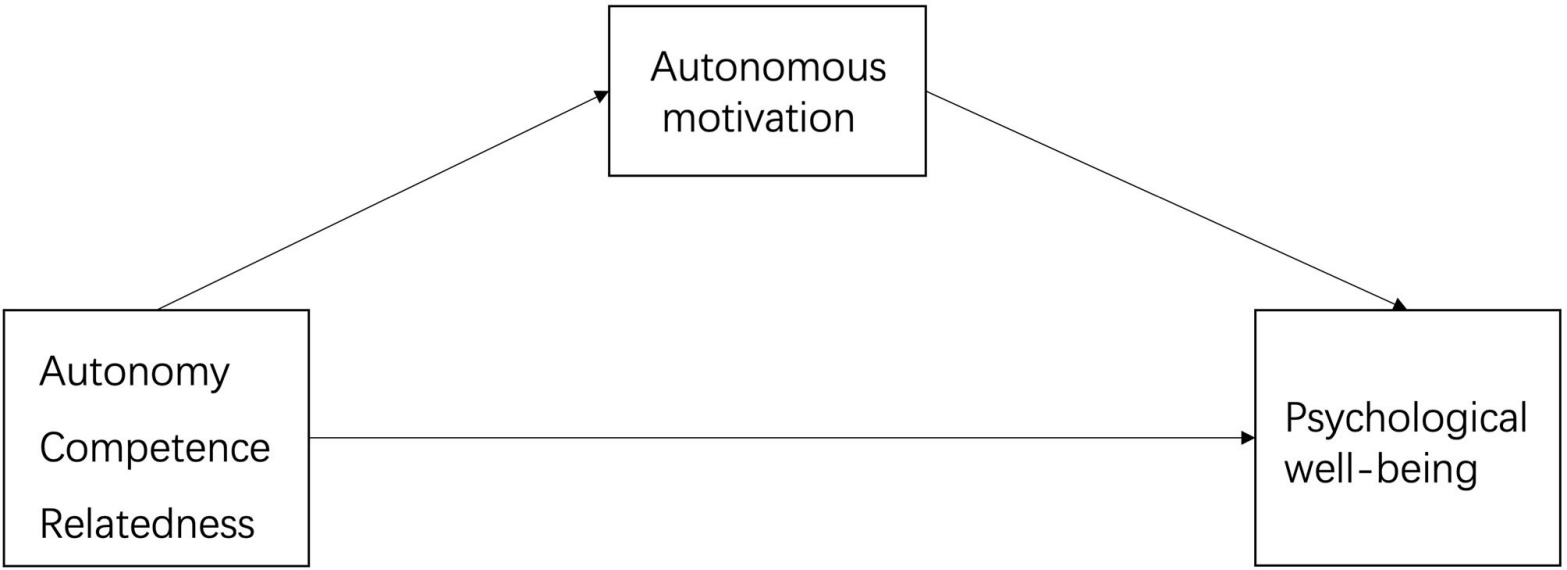
Self-determination theory showing hypothesized relations between basic psychological need satisfaction, autonomous motivation, and psychological well-being.

Finally, SDT posits that the basic psychological needs for autonomy, competence, and relatedness are innate and universal to all human beings, and the satisfaction of these psychological needs is essential for facilitating intrinsic motivation and playing a central role in energizing internalization of extrinsic motivation (controlled regulation to relatively autonomous self-regulation). Thus, when people are more successful at satisfying needs, they exhibit more autonomous motivation (intrinsic motivation and autonomous extrinsic motivation), because their behavior is more congruent with, rather than contradictory to, their own needs, goals, values, and interests, resulting in greater behavioral effectiveness and PWB. In summary, fulfillment of these individuals' basic psychological needs affords them the resources, nutrients, energy, or necessity to promote more autonomous self-regulation/autonomous motivation, all of which finally contribute to greater PWB (see model in Figure 1).

#### Previous Studies of Elderly Psychological Well-Being in the SDT Framework

Numerous empirical studies in various fields seem to have confirmed the perspective of SDT, including in the field of aging. According to a systematic review and meta-analysis on basic psychological need satisfaction, autonomous motivation, and PWB in later life (Tang et al., 2019a), results indicate that basic psychological need satisfaction and autonomous motivation are positively associated with PWB (life satisfaction, positive affect, adaptation, etc.).

In addition, three empirical studies examined in greater depth the relationship between needs satisfaction, motivation, and PWB. Specifically, a study conducted by Altintas et al. (2017) explored the relationship between participation in leisure activities, relatedness satisfaction, motivation, and adaptation among a group of French elderly people living in nursing homes. Their results suggested that satisfying basic needs (especially relatedness) enhances leisure participation, autonomous motivation, and adaptation to the nursing home environment. In fact, developing social experiences with peers, as well as feeling both connected to a group and integrated as an individual into that group (relatedness satisfaction), all help to enhance the elderly's leisure practice, improving autonomous motivation, and active participation in daily life, finally promoting better adaptive behaviors. Relatedness satisfaction has an indirect effect via autonomous motivation on people's adaptation in later life. Philippe and Vallerand (2008) designed a longitudinal study with a sample of older adults living in nursing homes in Canada to examine the role of the environment in changes in psychological adjustment over a 1-year period. Environments with more objective autonomy-support were associated with greater satisfaction of the basic need for autonomy, which was in turn conducive to higher autonomous motivation, and finally contributed to more positive changes in psychological adjustment 1 year later. Remarkably, autonomous motivation completely mediated the relationships between autonomy satisfaction and changes in psychological adjustment over a 1-year period. Their findings provide support for SDT and strongly suggest that needs satisfaction has an indirect effect on people's PWB (measured by satisfaction with life, self-esteem, meaning in life, and lack of depression) through autonomous motivation. In addition, Solberg et al. (2013) designed a 16-week longitudinal experiment to examine the relationship between needs satisfaction (especially competence satisfaction), motivation, and PWB (vitality), among elderly Norwegians in the context of participation in exercise. Their results also appear to support SDT's hypothesis: they found that the exercise intervention increased competence satisfaction and autonomous motivation, both of which affected a positive change in vitality over 16 weeks, highlighting the link between autonomous motivation and change in vitality through needs satisfaction (competence satisfaction).

Thus, three previous studies have examined the contribution of needs satisfaction for relatedness, autonomy, and competence, to autonomous motivation and PWB. Their findings all showed that both needs fulfillment and autonomous motivation positively influence elderly people's PWB; in fact, autonomous motivation mediated the relations between older people' needs satisfaction and their general PWB (e.g., adaptation, satisfaction with life, self-esteem, meaning in life, vitality).

Overall, the findings of previous research conducted in many different countries provide strong support for the SDT's view that needs satisfaction is universally important for facilitating autonomous motivation and promoting PWB for all individuals, regardless of cultural setting. There may be differences in the level of satisfaction of these basic needs arising from the individual sociocultural context, but the positive outcomes of needs satisfaction are not thought to be moderated by cultural backdrop.

### The Cross-Cultural Perspective

On the contrary, some cultural relativists reject the universal importance of autonomy in favor of the opinion that autonomy is simply a Western intellectual preoccupation rather than a universal concern, and hence lacks importance for Eastern peoples (e.g., Markus et al., 1996; Iyengar and DeVoe, 2003). In addition, they argue that relatedness is more significant for people from collectivist cultures but of less importance for Western peoples (e.g., Kitayama et al., 1997; Cross et al., 2002). Therefore, cultural differences should exist in the effects of autonomy satisfaction and relatedness satisfaction on motivation and PWB (Markus and Kitayama, 1991, 2003; Iyengar and DeVoe, 2003). More specifically, the influence of relatedness satisfaction on motivation and PWB should be strong in Eastern people while the impact of autonomy satisfaction on motivation and PWB should be greater in Western people.

Thus far, the cultural differences on the basic psychological needs are mainly reflected in two dimensions: (1) The level of perceived satisfaction of needs (e.g., Chirkov et al., 2005; Church et al., 2013); (2) assessment of the importance of the needs, especially with respect to PWB (e.g., Hahn and Oishi, 2006; Wang et al., 2007; Balkir et al., 2011). Specifically, a study conducted by Church et al. (2013) found that Asian participants (Japan, China, Malaysia, and the Philippines) averaged lower than non-Asian participants (U.S., Mexico, and Venezuela) in perceived satisfaction of autonomy and competence. The cultural differences in needs satisfaction found by this study were largely consistent with expectations from cultural psychology, and the authors explained their result as deriving from Asians' less independent self-construct. Furthermore, some studies have shown cultural differences in the strength of the relationship between basic psychological needs satisfaction and PWB, which were also more consistent with the cross-cultural perspective. Balkir et al. (2011) found that German women's (individualistic cultures) well-being was positively associated with autonomy satisfaction but not with relatedness satisfaction. Conversely, Turkish women's (collectivist cultures) well-being was positively associated with relatedness satisfaction but not with autonomy satisfaction. Another cross-cultural research conducted by Chen et al. (2016) found that although both Belgian (individualistic cultures) and Chinese adolescents (collectivist cultures) perceived the guilt-induction vignette to be more controlling than the autonomy-supportive vignette, this effect was more pronounced in Belgian adolescents. Similarly, Wang et al. (2007) reported that the beneficial effects of parents' psychological autonomy support (autonomy satisfaction) enhanced children's PWB (self-esteem, life satisfaction, positive emotion) and was generally stronger in the United States than in China. The authors explained this by theorizing that the United States has a prototypically independence-oriented culture which values autonomy, while China has a prototypically interdependence-oriented culture which favors collectivism. Autonomy would therefore, they proposed, be of more relevance in the United States than in China. Only one cross-cultural empirical study has been conducted in the elderly, and also indicated a cultural difference around needs satisfactions and the benefits of needs satisfaction to PWB (measured by positive and negative effects) comparing older adults in South Korea and American (Hahn and Oishi, 2006). Importantly, self-esteem was the most important need for older American adults, whereas self-actualizing-meaning (feeling a sense of deeper purpose in life) and popularity-influence (feeling that other people respect their advice) were the most important needs for older Korean adults. The authors explained this finding by suggesting that the Korean cultural tradition's emphasis of order and hierarchy in human relationships was at the root of this difference between their populations.

Overall, previous research conducted in different countries within the SDT framework in the field of aging, has proved the implication of needs satisfaction to autonomous motivation and well-being for elderly from different countries. These findings almost consistently confirm the SDT hypothesis. However, in regards to cultural differences in the universal importance of three aforementioned basic needs satisfaction (especially those of autonomy and relatedness) in Western and Eastern society, researchers have not yet reached a consensus conclusion, especially in the field of gerontology where research is still scarce. According to literature, previous research has focused heavily on the well-being of other populations (children, university students, adults, etc.). In the field of aging, strictly speaking, there has been no cross-cultural comparative research on the various benefits of needs satisfaction on well-being within the SDT framework. This oversight seems increasingly contradictory in light of the elderly world population being larger now than it has ever been in the past.

### The Present Study

As of today, elderly well-being is receiving increasing social attention, especially in countries like China where problems associated with aging are rapidly becoming more prevalent, and in which there are currently no comprehensive studies on elderly well-being within the SDT framework. China is the largest country of a predominantly Eastern culture. Unfortunately, the onset of China's psychological research occurred later than in Western countries; in particular, studies of psychology among the elderly were relatively few until the late 1990s. We are unaware of any published, empirical study conducted within the SDT framework among China's older population. We are also unaware of any international, cross-cultural studies in this field which includes elderly Chinese people. Thus, in this study, we sampled elderly from China and France. China, with a score of 20 for the measure of individualism, is thus considered to be a relatively collectivistic culture with a focus on values of interdependence, whereas France, with a score of 71, is more described as an individualist society (Hofstede, 2001; Chen et al., 2014).

The main purpose of this study is therefore, first to test the pertinence of the SDT framework among the elderly in China and in France, and then to examine the moderating effect of culture (Eastern vs. Western) on the relationship between needs satisfaction and autonomous motivation, as well as PWB. According to SDT, psychological needs satisfaction is essential for enhancing autonomous motivation and ongoing PWB (Ryan and Deci, 2000, 2017). We first tested hypothesis 1 that the satisfaction of three basic psychological needs is positively associated with autonomous motivation and PWB among elderly Chinese people. More precisely, the fulfillment of basic psychological needs predicts autonomous motivation and PWB, as well as autonomous motivation as a mediating variable, equally well among both elderly Chinese and French participants. With reference to the cross-cultural perspective that autonomy is less relevant for individuals living in Eastern cultures characterized by collectivistic values (rather than individualistic values) (Markus and Kitayama, 1991, 2003; Iyengar and DeVoe, 2003; Wang et al., 2007), we then tested hypothesis 2 that the benefits of autonomy satisfaction to PWB would be much stronger for the French sample; in contrast, the effect of relatedness satisfaction to PWB was expected to be higher among the elderly Chinese than the elderly French.

## Methods

### Participants^1^

Elderly Chinese data collection protocol was validated by the ethical committees of Beijing Normal University. Following the principle of strictly voluntary participation, one part of the data was collected via the university students conducting the questionnaire with their grandparents, while another part of the data was collected in leisure clubs for the elderly. All participants were retired and living at home. After deleting the cases with more than 8% missing data, 510 samples from Beijing were included consisting of 224 men and 286 women, with an age range of 50–97 years [50–59 years (7.5%), 60–69 years (52.1%), 70–79 years (33.1%), ≥80 years (8.3%), *M*_*age*_ = 68.49 years, *SD* = 6.88 years]. Education: 12.2% present with a primary school level of education, 32.4% a middle school level, 29.8% a high school level, and 25.7% a university level of education. Marital status: single (0.4%), married (84.7%), divorced (1.8%), widowed (13.1%). Concerning number of children: 2.4% had no children, 35.3% had one child, 42.7% had two children, 19.6% had three or more children. Living situation: 5.9% lived alone, 48.2% lived with a spouse, 17.8% lived with children, 26.7% lived with a spouse and children, while the rest constituted 1.4%. Average length of retirement was 13.39 years.

Elderly French data collection protocol was validated by the University of Lille's ethics committee. Following the principle of strictly voluntary participation, the same data collection methods were used as above with the retired, elderly participants in France. After rejecting cases with more than 8% missing data, a total of 170 older people (124 women and 46 men), also living at home and aged between 54 and 91 years [54–59 years (0.6%), 60–69 years (43.5%), 70–79 years (40%), ≥80 years (16.5%), *M*_*age*_ = 71.19 years, *SD* = 7.66 years], were recruited from Northern France. Education: 51.2% present a primary school level of education, 15.3% a middle school level, 5.9% a high school level, and 27.6% a university level of education. Marital status: single (4.1%), married (61.2%), divorced (8.2%), widowed (26.5%). Number of children: 4.1% had no children, 14.1% had one child, 31.2% had two children, 50.6% had three or more children. Living situation: 33.5% lived alone, 43.5% lived with a spouse, 2.4% lived with children, 18.2% with a spouse and children, and the rest represented 2.4%. Average length of retirement was 11.85 years.

### Measures and Procedures

#### Basic Psychological Needs Satisfaction

The Basic Psychological Needs Satisfaction Scale (BPNSS) for the elderly, from Indoumou Peppe et al. (2018), consists of 18 items divided into three dimensions: autonomy (e.g., “I generally feel free to express my ideas and opinions”), competence (e.g., “I feel competent in my activities”), and relatedness (e.g., “I respect and appreciate the people I meet”). Participants must answer each item on a five-point scale, ranging from 1 (not at all true for me) to 5 (completely true for me). The authors reported internal consistency indices varying between 0.78 and 0.83. According to SDT and previous studies (e.g., Custers et al., 2010; Mackenzie et al., 2017), global needs satisfaction was measured in both groups using the BPNSS, the average needs fulfillment across all items was computed with higher scores in general indicating greater needs fulfillment. In the current study, the Cronbach's alpha was 0.93 for the Chinese sample and 0.88 for French sample.

#### Motivation

The Elderly's Motivation Scale (EMS—Vallerand and O'Connor, 1991) consists of 72 items covering six domains of life: health, biological needs, interpersonal relationships, leisure, religion, and information. We removed questions about religion (12 items) in order to comply with French and Chinese legal particularities. Elderly's Motivation Scale quantifies four different motivational forms, including amotivation (e.g., “I don't know why I do it; I don't see what's in it for me”), controlled extrinsic motivation (e.g., “I do it because I am supposed to do it”), autonomous extrinsic motivation (e.g., “I choose to do it for my own good”), and intrinsic motivation (e.g., “I do it for the pleasure of doing it”). Participants answer each item on a seven-point scale, ranging from 1 (not at all) to 7 (exactly). The authors reported internal consistency indices ranging from 0.81 to 0.87. In accordance with SDT and previous studies, we estimated an autonomous motivation variable of the sum of the intrinsic motivation, and autonomous extrinsic motivation items (e.g., Williams et al., 2004; Solberg et al., 2013; Stupnisky et al., 2018). In the current study, the Cronbach's alpha was 0.95 for the Chinese sample and 0.92 for the French sample.

#### Psychological Well-Being

The PWBMMS of Massé et al. (1998) consists of 25 items that measure the dimensions of self-esteem (four items—e.g., “I felt confident”), balance (four items—e.g., “I felt emotionally balanced”), social commitment (four items—e.g., “I had goals and ambitions”), sociability (four items—e.g., “I had a lot of humor, I made my friends laugh”), self and events control (four items—e.g., “I was able to share things when faced with complex situations”), and happiness (five items—e.g., “I felt good about myself, at peace with myself”). Participants answer each item on a five-point scale, ranging from 1 (never) to 5 (almost always). The authors reported an overall Cronbach's alpha of 0.93 for the questionnaire, while in this study the Cronbach's alpha was 0.95 for Chinese sample and 0.91 for French sample.

#### The Linguistic and Cultural Translation

For the Chinese version of these questionnaires, original French scales were translated into Chinese by a bilingual person (first author of this study), which were discussed and revised with colleagues until a consensus was reached, including the grammatical structure and the choice of vocabulary (Chinese typically has a different basic sentence structure from French or English, and synonymy or polysemy often exists, hence we needed to choose a vocabulary familiar to older adults while maintaining semantic consistency). The back-translations were then performed by a French-Chinese language interpreter (a non-psychologist). Finally, a third person (the last author, whose native language is French), compared the original scales with the back-translated version and verified that the Chinese version had good internal consistency with the French original. Thereafter, these scales were deemed to have passed the comprehension evaluation step, and 10 elderly Chinese people were asked to answer the questionnaires and report any difficulty in comprehension. After translation, back-translation, and testing, the final version of the translated scales in Chinese was validated.

### Data Analysis

The Statistical Package for Social Sciences (IBM SPSS Statistical 20) and Mplus version 7.0 were used to analyze the data. Since the data were collected in two different countries, we tested for measurement invariance before performing SEM, and we reported the measurement invariance of all instruments after to preliminary analysis.

First, the independent *t*-test was performed with SPSS to verify whether there existed significant statistical differences between all study variables and demographic characteristics of the Chinese and French samples. Then, in each sample, the Pearson correlations were used to explore the association between satisfaction of basic psychological needs and motivation or PWB. For the main analysis, the structural equation modeling (SEM) was performed separately with Mplus 7.0 in two cultural samples, to estimate the path for the contribution of needs satisfaction to PWB, and examine whether motivation mediated the relation between needs satisfaction and PWB. The fit of the model was analyzed according to different criteria: the comparative fit index (CFI), the Tucker–Lewis index (TLI), the root means square error of approximation (RMSEA), and the standardized root mean square residual (SRMR). Finally, moderation analysis was performed in a full SEM with the overall elderly sample, to assess whether culture (Chinese vs. French) moderated the relationship between needs satisfaction, autonomous motivation, and PWB in our elderly populations. In regards to the small amount of missing data in the two samples, when the value of the variable was numeric (e.g., motivation, PWB), we effected single imputation (mean), meaning that the missing values were filled according to the average value of the rest of the sample. And for the missing, non-numeric values such as education, the missing values were filled with the highest occurrence value of the attribute in all other samples (Deng et al., 2019).

## Results

### Variables Characteristics of Two Samples

Table 1 presents the means and standard deviations of all the studied variables (basic psychological need satisfaction, motivation, and PWB) between the elderly Chinese and elderly French cultural groups. The results showed that the two samples did not present significant differences with respect to relatedness, global needs satisfaction, and autonomous extrinsic motivation. Elderly French people showed higher levels of autonomous satisfaction (*t* = −4.02, *p* < 0.001), intrinsic motivation (*t* = −13.40, *p* < 0.001), and autonomous motivation (*t* = −7.49, *p* < 0.001) than Chinese, while Chinese individuals presented higher levels for all the remaining variables such as competence (*t* = 3.62, *p* < 0.001), controlled extrinsic motivation (*t* = 5.31, *p* < 0.001), amotivation (*t* = 3.95, *p* < 0.001), PWB (*t* = 2.20, *p* < 0.05).

**Table 1:** Means and standard deviations of observed constructs in the Chinese and French sample.

	**China**		**France**				
**Variable**	***Min–Max***	***Mean ± SD***	***Mni–Max***	***Mean ± SD***	***Cohen's d***	***t*-test**	**95%CI**
Autonomy satisfaction	1.33–5	4.15 ± 0.72	2.33–5	4.35 ± 0.52	−0.32	*t* = −4.02, *p* < 0.001	[−0.32 −0.09]
Competence satisfaction	1.5–5	4.06 ± 0.71	2.33–5	3.87 ± 0.53	0.30	*t* = 3.62, *p* < 0.001	[0.08 0.29]
Relatedness satisfaction	1.17–5	4.25 ± 0.67	2.07–5	4.32 ± 0.48	−0.12	*t* = −1.49, *p* = 0.14	[−0.16 0.02]
Global needs satisfaction	1.39–5	4.15 ± 0.65	2.5–5	4.18 ± 0.44	0.05	*t* = 66, *p* = 0.51	[−0.12 0.06]
Intrinsic motivation	1–7	4.07 ± 1.53	2.37–7	5.38 ± 0.92	−1.04	*t* = −13.40, *p* < 0.001	[−1.51 −1.12]
Autonomous extrinsic motivation	1–7	5.96 ± 1.08	2.4–7	5.85 ± 0.93	0.11	*t* = 1.16, *p* = 0.25	[−0.07 0.29]
Controlled extrinsic motivation	1–7	4.18 ± 1.75	1–6.87	3.39 ± 1.65	0.46	*t* = 5.16, *p*. <0.001	[0.49 1.09]
Amotivation	1–7	2.25 ± 1.60	1–6.33	1.80 ± 1.17	0.32	*t* =3.95, *p* < 0.001	[0.23 0.68]
Autonomous motivation	1.17–7	5.01 ± 1.05	2.97–7	5.62 ± 0.86	−0.64	*t* = −7.49, *p* < 0.001	[−0.76 −0.44]
Psychological well-being	1.16–5	3.99 ± 0.66	2.21–5	3.88 ± 0.50	0.19	*t* = 2.20, *p* < 0.05	[0.01 0.20]

*N_China_ = 510, N_France_ = 170. CI, confidence interval; Mi, minimum range of variable; Max, maximum range of variable. Cohen's d, take the absolute value of Cohen's d, generally, a Cohen's d-value of 0.2–0.5 is a small effect, 0.5–0.8 is a medium effect, and a value above 0.8 is a large effect*.

### Relationship Between Basic Psychological Need Satisfaction, Motivation, and PWB

Table 2 shows the individual Pearson correlation coefficients for China (*r*_China_) and France (*r*_France_) between the study variables. The results indicated that the satisfaction of all the three needs (autonomy, competence, relatedness) showed significant positive correlations with autonomous motivation (including intrinsic motivation and autonomous extrinsic motivation) and PWB among the elderly in both China and France. Cultural differences were also observed: controlled extrinsic motivation was positively associated with global needs satisfaction (*r*_China_ = 0.18, *p* < 0.01) and PWB (*r*_China_ = 0.26, *p* < 0.01), while amotivation was found to be positively associated with intrinsic motivation and autonomous motivation only among Chinese elderly. Among the French elderly, amotivation was negatively correlated with autonomous motivation (*r*_France_ = −0.20, *p* < 0.01).

**Table 2:** Correlation coefficients of the study variables in the Chinese (lower half, rows) and French samples (upper, columns).

**Variables**	**1**	**2**	**3**	**4**	**5**	**6**	**7**	**8**	**9**	**10**
1 Autonomy satisfaction	1	0.67**	0.68**	0.90**	0.41**	0.55**	0.03	−0.13	0.52**	0.50**
2 Competence satisfaction	0.86**	1	0.60**	0.87**	0.45**	0.44**	0.1	−0.01	0.48**	0.51**
3 Relatedness satisfaction	0.75**	0.77**	1	0.86**	0.48**	0.52**	0.17*	0.03	0.54**	0.53**
4 Global needs satisfaction	0.94**	0.95**	0.90**	1	0.51**	0.57**	0.12	−0.04	0.58**	0.59**
5 Intrinsic motivation	0.16**	0.14**	0.14**	0.16**	1	0.72**	0.22**	−0.14	0.93**	0.42**
6 Autonomous extrinsic motivation	0.59**	0.56**	0.54**	0.61**	0.27**	1	0.12	−0.24**	0.93**	0.51**
7 Controlled extrinsic motivation	0.16**	0.18**	0.18**	0.18**	0.41**	0.33**	1	0.37**	0.18*	−0.02
8 Amotivation	−0.16**	−0.13**	−0.20**	−0.18**	0.43**	−0.14**	0.33**	1	−0.20**	−0.09
9 Autonomous motivation	0.42**	0.39**	0.38**	0.43**	0.87**	0.71**	0.47**	0.24**	1	0.50**
10 Psychological well-being	0.67**	0.71**	0.62**	0.71**	0.27**	0.58**	0.26**	−0.01	0.49**	1

*N_China_ = 510, N_France_ = 170*.

### Test Measurement Invariance

Before further analyses, we examined measurement invariance across countries for the latent measures included in the final model, in order to identify whether the concepts measured have the same meaning in the national samples included in our study. According to Chen (2008) and Byrne and Watkins (2003), measurement invariance was tested by the Multi-Group Confirmatory Factor Analysis (MGCFA). First, a configural invariance model was performed to test whether the loading pattern was similar in both groups. Second, a metric invariable or weak-invariance model was performed to test whether the factor loadings were equal in both samples, and the fit of this model compared to the configural invariance model. According to Byrne et al. (1989) and Cheung and Rensvold (2002), the Chi-square result and the analysis of changes in the comparative fit index (ΔCFI, the most widely-used and empirically best-supported criterion) were used to determine invariance at the measurement level. The results indicated that for the BPNSS, the differences in Chi-square were 16.82 (15), *p* > 0.05, and the change in CFI (ΔCFI = 0.001) and TLI (ΔTLI = 0.007) were less than the cut-off point of 0.01. For the EMS, the differences in Chi-square were 20.74 (16), *p* > 0.05, and the change in CFI (ΔCFI = 0.002) and TLI (ΔTLI = 0.002), also less than the cut-off point of 0.01. For the PWBMMS, the differences in Chi-square was 23.22 (16), *p* > 0.05, and the change in CFI (ΔCFI = 0.002) and TLI (ΔTLI = 0.002) again both less than the cut-off point of 0.01. To sum up, these measurement models exhibited measurement invariance in both of the studied samples.

### Common Method Bias Variance

When a research using the same method—especially the method of survey—to measure each variable, the common method variance (CMV) test is a routine yet important step in psychology, to determine whether the overlap of variation between two variables is caused by using similar measurement tools, and then to prove the true relationship between the latent variables (Tang and Wen, 2020). In this study, two popular statistical approaches were performed to examine the common-method variance.

First, Harman's single-factor test was performed to calculate the common-method variance of our study (Podsakoff and Organ, 1986). An exploratory factor analysis of all the items in the three questionnaires and the results showed that the first factor explained only 22.49 and 17.03% of the total variation in the Chinese and French populations, respectively—less than the critical value of 40%, and so the study could be considered to have no serious common-method variance (Zhou and Long, 2004; Tang and Wen, 2020).

Second, and after Podsakoff et al. (2003) and Tang and Wen (2020), a ULMC (unmeasured latent method construct) was performed to further assess the common-method variance in our two samples. Specifically, we included in the confirmatory factor analysis (CFA) model a common-method factor whose indicators included all the principal constructs' indicators, and then compared the fit of these two models (CFA vs. ULMC). The results demonstrated that the model fit index did not show significant improvement when including this common-method factor in our study. Specifically, the changes (Δ) in CFI and TLI were less than the critical value of 0.1, and the change in RMSEA and SRMR were less than the critical value of 0.05 (Wen et al., 2018) in both the Chinese and French populations, and we therefore concluded that there was no serious common-method variance in our study (ΔCFI = 0.04, ΔTLI = 0.03, ΔRMSEA = 0.02, and ΔSRMR = 0.03 for the Chinese sample, ΔCFI = 0.05, ΔTLI = 0.04, ΔRMSEA = 0.02, and ΔSRMR = 0.02 for the French sample).

### Analyses Examining Fit of the Mediation Model

To examine the contribution of each psychological need to autonomous motivation and PWB (how each psychological need influences autonomous motivation and PWB), we performed SEM in which PWB was established as the dependent variable and predicted by basic psychological needs satisfaction, while autonomous motivation was defined as a mediator between needs satisfaction and PWB. Previous studies have found that demographic factors such as gender, age, education, etc. (Altintas et al., 2017; Mackenzie et al., 2017) could have an impact on PWB, and preliminary *t*-tests revealed some significant differences of demographic characteristics between the Chinese and French samples. Thus, we controlled these variables in SEM by regressing PWB on them.

**Figure 2:**
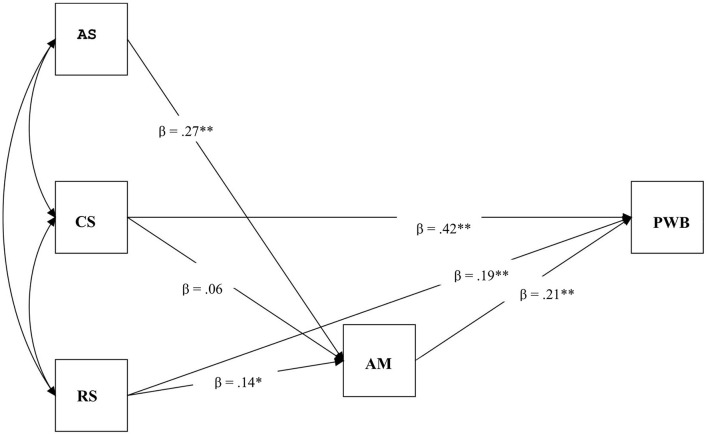
The path between needs satisfaction, motivation and PWB for the elderly Chinese. *X*^2^*/df* = 2.222, *p* = 0.014, RMSEA = 0.049, CFI = 0.979, TLI = 0.946, SRMR = 0.015. AS, autonomy satisfaction; CS, competence satisfaction; RS, relatedness satisfaction; AM, autonomous motivation; PWB, psychological well-being. *p < 0.05, **p < 0.01.

**Figure 3:**
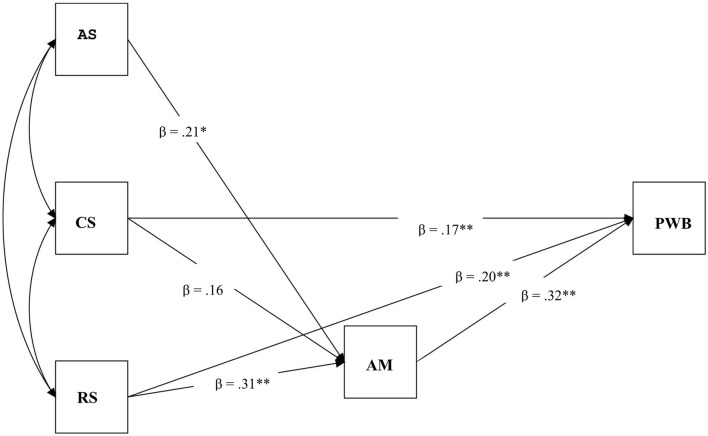
The path between needs satisfaction, motivation and PWB for the elderly French. *X*^2^*/df* = 1.489, *p* = 0.136, RMSEA = 0.054, CFI = 0.970, TLI = 0.925, SRMR = 0.022. AS, autonomy satisfaction; CS, competence satisfaction; RS, relatedness satisfaction; AM, autonomous motivation. *p < 0.05, **p < 0.01.

In accordance with our first hypothesis, SEM revealed the same path in both the Chinese and French samples. As can be seen in Figures 2, 3, the chi-square statistics were not significant in either sample and other fit indices also indicated a good fit of the structural model in both countries. For China, X^2^/*df* = 2.222, *p* = 0.014, the CFI was 0.979, the TLI was 0.946, RMSEA was 0.049, and the SRMR was 0.015; For France, X^2^/*df* = 1.489, *p* = 0.136, the CFI was 0.970, the TLI was 0.925, RMSEA was 0.054, and the SRMR was 0.022. All paths shown in Figures 1, 2 were significant in both samples except the path from competence satisfaction to autonomous motivation. Overall, these analyses indicate that the model fits the data satisfactorily in both samples: one path was that basic psychological needs satisfaction (more precisely, competence satisfaction, and relatedness satisfaction) directly enhanced PWB; at the same time, another path was that basic psychological needs satisfaction (more precisely, autonomy satisfaction and relatedness satisfaction) enhanced autonomous motivation, which in turn promoted PWB, autonomous motivation accounted for a partial mediation effect between needs satisfaction and PWB among the elderly in both samples. Therefore, our first hypothesis was supported.

### Moderation Model: The Moderating Effect of Culture

Next, to examine the moderating effect of culture, we performed a full SEM with the overall elderly sample, which treated culture as a moderator on the model. More precisely, we conducted moderation analysis using Mplus 7.0 with 1,000 bootstrap samples (Hayes, 2012) to examine whether belonging to an Eastern or Western culture (Chinese vs. French elderly) moderated the effect of needs satisfaction on autonomous motivation and PWB.

As can be seen in Table 3, the results did not support the supposition that culture acts as a moderator in the relationship between autonomy satisfaction and autonomous motivation (estimate = 0.02, *p* = 0.89), between relatedness satisfaction and autonomous motivation (estimate = 0.38, *p* = 0.06), between relatedness satisfaction and PWB (estimate = 0.16, *p* = 0.20), or between autonomous motivation and PWB (estimate = 0.02, *p* = 0.75). However, we did observe that culture moderated the effects of competence satisfaction on PWB, more precisely, the contribution of competence satisfaction to PWB was stronger among elderly Chinese than elderly French people (estimate = 0.23, *p* < 0.05) (see Table 3).

**Table 3:** The moderating effect of culture (China/France) on the structural pathway coefficient between needs satisfaction, autonomous motivation, and PWB.

**Pathway**	**Estimate**	**SE**	***p*-value**	**95%CI**
AS → AM	0.02	0.17	0.89	[−0.34 0.33]
RS → AM	0.38	0.20	0.06	[−0.79 0.02]
CS → PWB	0.23	0.11	<0.05	[0.04 0.46]
RS → PWB	0.16	0.12	0.20	[−0.39 0.06]
AM → PWB	0.02	0.06	0.75	[−0.09 0.14]

*N_China_ = 510, N_France_ = 170; AS, autonomy satisfaction; CS, competence satisfaction; RS, relatedness satisfaction; AM, autonomous motivation; PWB, psychological well-being*.

Simple slope analysis was used to further verify the interaction effects. Results of a simple slope test showed that the effect of competence satisfaction was significant for elderly French (simple slope = 0.48, *t* = 7.15, *p* < 0.001). However, the simple slope test for elderly Chinese showed a stronger effect of competence satisfaction on PWB (simple slope = 0.65, *t* = 22.82, *p* < 0.001).

## Discussion

The present study tested the mediation model of SDT as applied to the field of aging in both China and France. It then directly compared the benefits of needs satisfaction for autonomous motivation and well-being in individuals from China (where collectivistic values are more prominent) and in individuals from France (where individualistic values are more prominent).

The findings supported our hypothesis 1 stating that basic psychological needs satisfaction relates to autonomous motivation and PWB, as well as that autonomous motivation mediates the relation between needs satisfaction and PWB, equally well among both elderly Chinese and French participants. However, our finding did not support our hypothesis 2. This means that the relation from autonomy satisfaction to PWB was not stronger in the French sample, and the effect from relatedness satisfaction to PWB was not stronger among the elderly Chinese. Interestingly enough, however, we found a significant moderating effect of culture in the relation between competence satisfaction and PWB, in that the contribution of competence satisfaction on PWB being was stronger among elderly Chinese than elderly French.

Self-determination theory posits that the satisfaction of the needs for autonomy, competence, and relatedness is a key factor in promoting autonomous motivation and PWB, across age and cultures. In contrast, this generalization of human needs across cultures is often challenged by cross-cultural psychologists, who doubt that there are universal psychological needs for individuals from all cultures (Heine et al., 1999).

Accordingly, the present study was intended, in part, to examine the universality of the SDT framework by comparing older Chinese people with older French people, thereby comparing cultures influenced by Eastern collectivist culture and Western individualistic culture, respectively. To our knowledge, this remains a field completely lacking in empirical data collected within the framework of SDT. Our findings indicated that positive correlations between autonomous motivation (intrinsic motivation and autonomous extrinsic motivation) and PWB among the elderly Chinese, which are generally consistent with SDT, are similar to our findings from French participants as well as the results from previous studies conducted within Western countries (Tang et al., 2019a). This means that the older adults with high levels of need satisfaction show higher levels of autonomous motivation and PWB, whether from China or France. This finding provides evidence for the pertinence of SDT for studies among Eastern cultures, and more specifically, in the field of the psychology of older Chinese people.

More particularly, this study then tested a self-determination model of PWB among the healthy elderly living at home, with perceived satisfaction of three needs being theorized to facilitate autonomous motivation, and in turn being hypothesized to promote PWB. The model, supported by previous studies in France, was examined using data from older adults living at home or in nursing homes (Ferrand et al., 2012; Altintas et al., 2017, 2018). Analyses revealed that the fit indices indicated a good fit of the structural model for the cross-cultural sample, providing general support for the universality of SDT. The satisfaction of autonomy and relatedness predicted autonomous motivation in both elderly Chinese and French, and the degree of autonomous motivation in turn predicted their PWB, while autonomous motivation accounted for a partial mediation effect between needs satisfaction and PWB among the elderly in both countries. Thus, by showing that satisfying these needs promotes autonomous motivation and PWB across cultures, results from our study confirmed that the SDT model of PWB was universal, regardless of culture.

Finally, to examine whether Eastern or Western culture (Chinese vs. French) moderated the effect of needs satisfaction on autonomous motivation and PWB, we conducted an analysis using country as a moderating variable in the structural model. Results indicated no moderating effect of culture on the relationship between basic psychological needs satisfaction (except competence satisfaction), autonomous motivation, or PWB. This means that the importance of satisfying three basic needs to PWB were equivalent across the two countries. More specifically, the beneficial effect of autonomy satisfaction to the elderly's PWB was not higher in France than in China, and similarly the benefits of relatedness satisfaction to PWB were not stronger among the Chinese than among the French. These results are inconsistent with the opinion of self-construal theory (Markus and Kitayama, 1991), and by contrast, these findings provide further evidence in support of the universality of the SDT's hypotheses, which propose that these needs are universal (Deci and Ryan, 1985; Ryan and Deci, 2017).

Interesting cultural differences were nevertheless observed with regard to the following: firstly, concerning the level of participants' perceptions of needs satisfaction, in particular of the satisfaction of autonomy and competence, French people showed higher levels of autonomy satisfaction than Chinese people. This is consistent with the study of Church et al. (2013), which indicated occidental participants (members of individualistic cultures) averaged higher than Asian participants (members of collectivistic cultures) in perceived satisfaction of autonomy. This may be explained by the fact that individuals living in an individualist environment typically feel less obliged to follow the dictates of others and have more opportunity to perceive autonomy through their autonomous behaviors. However, Chinese individuals presented higher levels of competence satisfaction, which better predicted the PWB among elderly Chinese than French. This is inconsistent with the findings of Church et al. (2013), but may be explained by the location of recruitment of Chinese participants. 510 elderly Chinese were recruited from Beijing, the capital of China, where people often have a higher level of education, which is positively associated with perceived health (Abolfathi Momtaz et al., 2014), and which in turn influences individuals' needs satisfaction.

Our second cross-cultural finding is in regard to motivation. Older Chinese adults exhibited a lower level of intrinsic motivation, which was positively correlated with amotivation when compared with their French counterparts. This is not in line with the hypothesis of SDT, nor is it consistent with the findings from previous studies in Western populations, in which there was a negative correlation between amotivation and intrinsic motivation (Losier et al., 1993; O'Connor and Vallerand, 1994; Altintas et al., 2017), or else a lack of statistically significant correlation (Solberg et al., 2013; Altintas and Guerrien, 2014). These results may be explained by the fact that the elderly in China are deeply influenced by a collectivist culture. Compared with the French elderly, who are influenced by an individualistic culture, Chinese elderly may be more likely to ignore their own internal interests, thus showing that the level of intrinsic motivation is significantly lower than that of the elderly in France. On the other hand, older Chinese adults' level of amotivation was significantly higher than that of the elderly in France, which may be explained by the strong influence of “modesty” in classical Chinese philosophy, meaning that Chinese individuals generally pay more attention to other people's perspectives or social evaluations. Therefore, for certain questions, their answers are usually modest; otherwise, they may choose not to answer the question, or reply “I don't know” so as to avoid being questioned. In terms of SDT, these behavioral traits could result, on the one hand, in a lower level of intrinsic motivation, and on the other hand, in a higher level of amotivation. This may explain why the two types of motivation were statistically positively correlated (Tang et al., 2019b).

Finally, cultural differences were observed with regard to the positive association between the needs satisfaction and controlled extrinsic motivation: a significant association between controlled extrinsic motivation and PWB existed only among the elderly Chinese people, but was absent in the French population. This is inconsistent with the hypothesis of SDT, and also inconsistent with the findings from previous studies in Western populations (O'Connor and Vallerand, 1994; Vallerand et al., 1995; Solberg et al., 2013), in which controlled extrinsic motivation was negatively associated with PWB indices such as psychological adjustment, subjective PWB, and life satisfaction. This could be due to the fact that people living in an individualistic culture may see pressure or obligation as externally imposed constraints, while people living in a collectivist culture may instead see them as useful supports that serve the needs of the society (Lepper et al., 2005). It is perhaps not surprising, therefore, that Chinese people are more likely to follow external norms, like Confucius, who concluded that he had been able to do what he intended freely while conforming to norms since the age of 70 years (cong xin suo yu, bu yuju; The Analects, Chapter 2, Verse 4). This may explain why there was a positive association between needs satisfaction, controlled extrinsic motivation and PWB among the elderly Chinese, and means that they can respond to external pressure or obligation without decreasing their level of PWB.

It should be noted that this study has some limits. First among these is the unequal sample size: equal-sized groups maximize statistical power, and having unequal sample sizes may have affected either the number or level of significant results (Rusticus and Lovato, 2014). In fact, as we mentioned several times before, this study seems to be the first application of the SDT on an elderly Chinese population; there is thus a paucity of data, examining the capacity of these scales to provide accurate psychometric measurements in Chinese populations. Thus, this research needs firstly to ensure that the instruments used are culturally viable, which is a crucial step in maintaining the validity of data. Additionally, in regards to the estimated sample size for a revising scale, previous literature has generally been based on the rule of five to ten participants for each item, or the rule of a minimum of five participants for each item. In view of the tools used in the present study, we required 300–600 Chinese subjects; this, however, led to the Chinese sample being larger than the French sample. This incongruency could then be said to have introduced the problem of an unequal sample size. Therefore, further research on this issue will be necessary to achieve generalizable results. The second limitation is related to the sociodemographic differences: there are some differences in the levels of age, sex, education, marital status, number of children, living situation, and length of retirement between the two groups. Although we have controlled for these variables during statistical analyses, these differences may still have an impact on our interpretation of the results. Finally, in this study we did not directly measure culture-related variables, such as the level of individualism of each participant, so as to shed light on the measurable cultural differences between the participating countries.

## Conclusion

Our findings confirmed the universality of SDT and hence its applicability to the Chinese and the French population (first time for the Chinese elderly), specifically in the field of the psychology of aging. More specifically, basic psychological needs satisfaction relates to autonomous motivation and PWB, and autonomous motivation was found to mediate the relation between needs satisfaction and PWB as a mediating variable. Consistent with SDT, cultural differences were found in the degree of needs satisfaction, but much of the strength of the relationship between needs satisfaction (autonomy and relatedness) and autonomous motivation or needs satisfaction and PWB were identical. On the other hand, the data also highlighted the importance of attending to cultural differences in the prediction of competence satisfaction's contribution to PWB, and in the correlation between motivation and PWB.

## Data Availability Statement

The raw data supporting the conclusions of this article will be made available by the authors, without undue reservation.

## Ethics Statement

The studies involving human participants were reviewed and approved by Beijing Normal University (Ethical Committee reference n°20161221) and University of Lille (Ethical Committee reference n°2017-3-S48). The patients/participants provided their written informed consent to participate in this study.

## Author Contributions

MT, DW, and AG were responsible for the developmentand implementation of the study protocol, analysis, and final writing of this report. All authors contributed to the article and approved the submitted version.

## Conflict of Interest

The authors declare that the research was conducted in the absence of any commercial or financial relationships that could be construed as a potential conflict of interest.

## Publisher's Note

All claims expressed in this article are solely those of the authors and do not necessarily represent those of their affiliated organizations, or those of the publisher, the editors and the reviewers. Any product that may be evaluated in this article, or claim that may be made by its manufacturer, is not guaranteed or endorsed by the publisher.

## Footnotes

^1^Aging is defined by the WHO as a process of gradual change in the body's biological, psychological, and social structures and functions, resulting from the passage of time. While aging is an individual process, old age is the condition that characterizes a particular age group (Fontaine, 2007). If we ask ourselves at what point a person enters old age, it is possible to affirm that there is no universal definition of an elderly person: so should we consider the chronological age, physiological age, or determine age based on events like retirement? From an economic or social perspective, we refer to the age of cessation of professional activity, corresponding to the time of retirement as a transition into old age (Caradec, 2008). In many countries, the retirement age is set at 65; and it is for that reason that this age is considered the turning point between adulthood and old age. However, the age of retirement varies by country. In France, it is generally 62 years; in China, there are variables according to gender and professional category: 60 years for men, 50 for employed women, 55 years for female executives. Therefore, in order to maintain the consistency between the two cultural samples, all the subjects in this study were collected at least 50 years of age and represented the status of retired.
